# Food taboos and associated factors among agro-pastoralist pregnant women: A community-based cross-sectional study in Eastern Ethiopia

**DOI:** 10.1016/j.heliyon.2022.e10923

**Published:** 2022-10-05

**Authors:** Tesfa Mengie, Yadeta Dessie, Gudina Egata, Temesgen Muche, Samuel Derbie Habtegiorgis, Lemma Getacher

**Affiliations:** a Amhara Regional Health Bureau, CDC Project, Monitoring and Evaluation Officer, Dessie, Ethiopia; b School of Public Health, College of Health and Medical Sciences, Haramaya University, Harar, Ethiopia; c School of Public Health, College of Health Science, Addis Ababa University, Addis Ababa, Ethiopia; d Department of Nutrition, School of Public Health, College of Health Science, Dilla University, Dilla, Ethiopia; e Department of Public Health, College of Health Science, Institute of Medicine and Health Science, Debre Markos University, Debre Markos, Ethiopia; f School of Public Health, Asrat Woldeyes Health Science Campus, Debre Berhan University, Debre Berhan, Ethiopia

**Keywords:** Food taboos, Pregnant women, Ethiopia, Prevalence, Agro-pastoralist area

## Abstract

In underdeveloped nations, cultural norms that are harmful to women's health, such as food taboos, are responsible for five to fifteen percent of pregnancy-related deaths. Food Taboo traditions prevent women from consuming particular foods, which reduces dietary diversity and food quality and may have detrimental nutritional effects. However, little is known about Ethiopia’s dietary taboos and related issues. So, the purpose of this study was to find out how common food taboos are among pregnant women in agro pastoralist settings, as well as the accompanying factors. 636 pregnant women were enrolled in a community-based cross-sectional study using a two-stage cluster sampling strategy, distributed over seven clusters. Data were exported from Epi Data version 3.01 to Statistical Package for Social Science version 20 after being entered. The prevalence of dietary taboos in this study was 67.4% (95% CI: 63.7%, 71.1%). Food taboos were independently and significantly predicted by lack of formal education [AOR = 1.97 (95% CI: 1.583, 4.496), low wealth index [AOR = 2.26 (95% CI: 1.173, 4.353)], absence of antenatal care visits [AOR = 6.16 (95% CI: 4.996, 10.128), lack of knowledge of maternal nutrition [AOR = 4.94 (95% CI: 3.799, 8.748)], and negative attitude toward maternal nutrition [ In the research area, dietary taboos were very common. Food taboos were independently predicted by low wealth index, lack of maternity care visits, lack of formal education, ignorance of maternal nutrition, and unfavorable attitudes. Therefore, it is highly advised that strong community-based maternal nutrition education and counseling, raising women's income, and preparing young women for study in order to improve their educational standing be implemented.

## 1 Introduction

Food taboos are dietary laws that allow or forbid particular foods in a particular culture, group, or community. They frequently coincide with noteworthy occurrences or stages of the human life cycle as illness, menstruation, pregnancy, and lactation. Malnutrition is said to be primarily (and indirectly) caused by food taboos. This food restriction could deplete women’s bodies of essential nutrients and have a generational impact on the health of their offspring. The eating of food derived from animals is commonly prohibited, especially among racial and ethnic groups who frequently lack protein. Food prohibitions connected to pregnancy are dietary guidelines in a particular culture that forbid certain foods when pregnant [1, 2].

Certain foods are off-limits to women due to food prohibitions. Thus, restricting dietary diversity and quality may have negative effects on one’s health and nutritional status. Every culture has a different level of practice and different foods that are avoided. However, compared to urban and better educated populations, rural and less educated cultures tend to have a higher prevalence of food aversions. Women who are pregnant observe food restrictions more closely than women who are not pregnant [2, 3, 4, 5].

Due to the physiological increase in nutrient demand during pregnancy, which may not be sufficiently supplied by dietary consumption, pregnancy alone puts pregnant women prone to malnutrition. Therefore, further dietary restrictions brought on by pregnancy-related food restrictions and beliefs may negatively impact both the mother's and the fetus' health [6, 7].

Pregnant women’s nutritional status can be affected by food accessibility, food availability, maternal knowledge, attitude, and perception of various foods. It is crucial to consume foods that are nutritionally balanced while expecting. However, cultural taboos, norms, and religious beliefs have a significant impact on the eating behaviors of pregnant women [8].

Women, whether they live in the country or the city, each have their unique food prohibition beliefs and customs. To fulfill the mother’s increased needs and avoid nutritional deficiencies, a balanced and sufficient diet is crucial throughout pregnancy [9]. Key protein sources like meat, eggs, and fish were the most popular diets, according to a study conducted in India [10].

To fulfill the increased nutritional needs of the mother and the fetus as well as to prevent nutritional deficiencies, a balanced and sufficient diet is crucial during pregnancy. Pregnancy and delivery outcomes are impacted by inadequate maternal nutrition, particularly in rural areas. Women may lack an acceptable nutritional status due to the avoidance of specific foods, poor access to and availability of food, along with inadequate knowledge of its benefits. Food restrictions have been named as one of the causes of maternal undernutrition in pregnancy, particularly in Ethiopia’s rural areas [4].

According to a number of evidences, women who experienced problematic pregnancies and greater rates of cesarean sections adhered to their food aversions, yet pregnant women's beliefs run counter to this conclusion [4, 5, 6, 7, 8]. Despite the fact that food-related issues are widespread in Ethiopia, little is known about them. This study, which involved a pastoralist community, is the first of its sort to be carried out in Eastern Ethiopia, to the best of the researchers' knowledge. Therefore, the purpose of this study was to find out how common food taboos are among pregnant women in Eastern Ethiopia and to pinpoint the contributing causes.

## 2 Materials and methods

### 2.1 Setting period and study design

From February to March 2017, the research was carried out in the eastern part of Ethiopia, in the Gursum district. Gursum district is found in the Fafan zone of Somali Regional State, which is located 554 km east of Addis Ababa in Ethiopia. It is located at 90 20N″ latitude and 42,035″ E longitude, and its elevation ranges from 1200 to 2950 m above sea level. The district has 15 kebeles and a total population of 73,038 people, with 38,044 males and 34,994 females, and 4820 households. There were 1478 pregnant women among the total population of the district. The district has 2 health centers, 15 health posts, and 56 health professionals.

A community-based cross-sectional study design was employed with 636 pregnant recruited in seven clusters. These samples were selected by two-stage cluster sampling and all pregnant women in the selected clusters were interviewed. Data were entered into Epi-Data version 3.01 and exported to Statistical Package for Social Science (SPSS) version 20 for further analysis.

### 2.2 Population

The study's source population included all pregnant women in the district. The study population, on the other hand, consisted of all pregnant women in randomly selected kebeles (the smallest administrative unit in Ethiopia's political structure). In terms of inclusion criteria, all pregnant women who had lived in the district for at least six months were eligible. Pregnant women who were seriously ill during the study period, on the other hand, were excluded.

### 2.3 Sample size determination and sampling procedures

The sample size for this study was calculated using the following assumptions: the prevalence of food taboos (27%) [3] the margin of error (d) = 5%, the standard normal deviate (Za/2) = 1.96 corresponds to a 95% confidence level, 5% for nonresponse rate, and a design effect of 2. Accordingly, the final sample size was 636.

Regarding the sampling procedure, pregnant women were selected using a cluster sampling technique. The district has 15 kebeles in total. All kebeles of the district were clustered in to urban (2 kebeles) and rural (13 kebeles). From each category, kebeles were selected by simple random sampling using a lottery method. To get the required number of pregnant mothers, proportional allocation was used for both rural (540) and urban (98) sites. At the end, through the house-to-house visit, all pregnant women in randomly selected clusters were included in the study.

### 2.4 Data collection methods and instruments

A structured interviewer administered questionnaire was prepared which was adapted from previous studies to collect the data for this study [11, 12]. By modifying the list of food items, the knowledge and attitude related questionnaires were adapted from FAO 2014 [1].

### 2.5 Measurement

Maternal knowledge on maternal nutrition during pregnancy was assessed to assess the level of knowledge of women about nutrition during pregnancy. It was measured based on five knowledge assessment questions by giving 1 score for an appropriate answer and 0 score for inappropriate answers. After checking the normality, the mean score was taken as a cut point. Participants with a mean score (50%) and above were considered as having good knowledge, while those scored less than the mean score (50%) were considered as having poor knowledge [13].

Attitude towards maternal nutrition during pregnancy was assessed to explore pregnant women beliefs and level of agreement with maternal nutrition during gestation. It was measured based on 5 attitude assessment questions using a 5-point Likert scale. All of these questions were scored from 1 to 5 (1 = strongly disagree, 2 = disagree, 3 = neutral, 4 = agree, and 5 = strongly agree) After checking the normality, the mean score was taken as a cut point then those participants with a mean score (50%) and above were considered as having a favorable attitude towards maternal nutrition while those scored less than the mean score (50%) were considered as having unfavorable attitudes [13].

The practice of food taboos (outcome variable) during gestation was assessed to examine the unusual prohibition of foods due to either cultural or religious food beliefs. It was measured based on a question which directly asks the presence or absence of an unusual food prohibition for at least one food item during pregnancy by using a yes or no question. During coding, 1 was given for yes while 0 was given for no answer. Then the result was expressed using percentage and before logistic regression analysis it was recorded in two different variables as yes (outcome variable) = 1 and no = 0. Number of food items prohibited and their respective reasons were measured and expressed in percentage of data answered yes for the food taboo practice question [13].

### 2.6 Data quality control

The English version questionnaire was translated to Af- Somali (the local language). To ensure its consistency, translating back to English language was done. All data collectors, facilitators, and supervisors were trained for two days before the actual date of data collection on the purpose of the study, content of the questionnaire and interview guides, interviewing methods, confidentiality, interpersonal communication and other relevant issues to the study. There was a pretest before collection of data in Goljano kebele which have no geographical and cultural variation on 5% of the sample. This was not included in the analysis but for questionnaire modification only. The entire questionnaire was checked daily through close supervision. All data collectors were female trained nurses as it has the advantage to minimize social desirability bias since culture is sensitive. To keep consistency in data entry, double data entry was done. Regression analysis and multicollinearity diagnosis was performed.

### 2.7 Data processing and analysis

The entire questionnaire was cross-checked before data entry by the principal investigator. Data were entered into Epi-Data version 3.01 and exported to SPSS version 20 statistical software for analysis. Chi-square test was done to identify the association of dependent and independent variables. Variables that showed association with the outcome variable (p-value = 0.3) during bivariate logistic regression analyses were entered to multivariate logistic regression. Multicollinearity was checked using standard error. Variables with a standard error greater than 2 were dropped from multivariate analysis. Hosmer Lemeshow goodness of model fit test was done to test for the model fitness at p > 0.05. Odds ratio along with 95% confidence interval was computed using multivariable logistic regression to control for the effect of confounding variables. Level of statistical significance was declared at a p value less than 0.05 and all tests were two-sided. Results were presented using texts, tables, and figures.

## 2.8 Ethics approval

The ethical approval was obtained from the College of Health and Medical Science Institutional Health Research Ethics Review Committee (IHRERC) of Haramaya University. It was dated 05 December 2017 and numbered with Ref C/AC/R/D/792/17. The clear description of the study title, purpose, procedure, duration, possible risks, and benefits of the study was explained for each study participant and obtained before informed consent. Then, written informed consent was obtained from each respondent before starting the interview.

## 3 Results

### 3.1 Sociodemographic characteristics of study participants

The sociodemographic characteristics of the study participants are shown in Table 1. In this study, the response rate was 95.9%. The mean age of study participant was 32 (SD ± 7 years). Of the participants, 45.73% were above 34 years old, 89.3% rural in residence, 92.1% Somali in ethnicity, 85.1% married in marital status, 93.6% were Muslim in religion and 72.46% of the participants had no formal education (Table 1).

**Table 1:** Distribution of sociodemographic characteristics of the study participants in 2017.

Variable	Frequency (n = 610)	Percentage (%)
**Residence**		
Rural	512	83.9
Urban	98	16.1
**Age**		
15–24	116	19.02
25–34	215	35.25
>34	279	45.73
**Ethnicity**		
Somali	562	92.1
Oromo	27	4.4
Others∗	21	3.5
**Religion**		
Muslim	571	93.6
Orthodox	22	3.6
Protestant	17	2.8
**Marital status**		
Married	519	85.1
Divorced	70	11.5
Others∗∗	21	3.4
**Educational status**		
No formal education	442	72.46
Formal Education	168	27.54
**Occupation**		
Housewife	77	12.6
Gov't employer	61	10.0
Merchant	48	7.9
Others∗∗∗	199	32.6
**Wealth Index** low		
Medium	208	34.1
High	203	33.3

∗ = Amhara and Gurage; ∗∗ = never married, separated, widowed, ∗∗∗ = daily laborers, NGO worker.

### 3.2 Reproductive history of pregnant women

Related to the reproductive history of the study participants, three-fifth (59.5%) of the participant had no antenatal care visits, while only 1.6 % had completed fourth antenatal care visits. More than half of the participant (56.2%) were grand multigravida and (45.4%) were multipara (Table 2).

**Table 2:** Distribution of reproductive history of pregnant women in Gursum district, Eastern Ethiopia in 2017.

Variables	Frequency	Percentage (%)
Previous ANC visits		
Yes	247	40.5
No	363	59.5
Gravida		
Primi	363	59.5
Multi	67	11.0
Grandmulti	200	32.8
Para		
Nullipara	343	56.2
Primipara	89	14.6
Multipara	277	45.4
Grandmulti	177	29.0

### 3.3 Prevalence of food taboos among pregnant women

In this study, the prevalence of food taboos was 67.4% (95% CI: 63.7%, 71.1%). The findings of this study showed that the most avoided food item was meat which was avoided by 67.4% of the study participants, followed by egg, carbonated drinks, pasta with sauce and milk, 66.2%, 58.5%, 56.4% and 36.6%, respectively. The most commonly reasons for the increased food taboos were fear of difficult delivery resulted from a large size fetus which was reported by about 67.4% of the study participants followed by fear of abortion (62.1%) and fear related to plastered fetal head (36.6%) (Table 3).

**Table 3:** Distribution of each food item prohibited and their respective reasons during pregnancy in Gursum, Ethiopia, in 2017.

Tabooed Food Item	Frequency (%)	Reasons for tabooed food items
Carbonated drinks	357 (58.5)	Fear of abortion as a result of increased fetal movement
Meat	411 (67.4)	Fetus become big and will cause prolonged and difficult labor & delivery
Egg	403 (66.2)	Fetus become big and will cause prolonged and difficult labor & delivery
Milk	224 (36.6)	Plastered fetal head
Pasta with sauce	344 (56.4)	Fear of abortion as a result of increased fetal movement
Rice	261 (42.7)	Fear of abortion as a result of increased fetal movement
Banana	342 (56.3)	Fear of abortion as a result of increased fetal movement
Papaya	369 (60.5)	Fear of abortion as a result of increased fetal movement
Mango	226 (37.2)	Fetus become big and will cause prolonged and difficult labor & delivery
Cold Water	336 (55.1)	Will increase fetus cephalic size and cause malformation and cause prolonged and difficult labor
Others∗∗	208 (34.1)	Fear of abortion as a result of increased fetal movement
Food taboos	Yes 411 (67.4%)	Prevalence of food taboos
	No 199 (32.6%)	

∗ = and ∗∗ = lemon, honey, hot porridge.

### 3.4 Knowledge and attitude of women about maternal nutrition during pregnancy

This study found that nearly two-third (65.3%) of participant had poor knowledge of maternal nutrition and more than three-fifth (62.5%) of participants had negative attitudes towards maternal nutrition.

### 3.5 Prohibited food items and their respective reasons

Meat was the most prohibited food with a reason of the fetus becoming big and will cause prolonged and difficult labor and delivery (Table 3).

### 3.6 Factors associated with food taboo

In this study, in the multivariate logistic regression analysis, variables such as having no formal education, having low wealth index, not having antenatal care visits, having poor knowledge about maternal nutrition, and having negative attitude towards maternal nutrition showed a significant association with food taboos (Table 4).

**Table 4:** Bivariate and multivariate analysis of factors associated with food taboos among pregnant women in 2017.

Variables	Food taboos		COR (95%CI)	AOR (95%CI)
	Yes (%)	No (%)		
**Women's age**				
15–24	108 (17.71)	8 (1.31)	2.04 (1.58, 4.35)	1.27 (0.07, 3.71)
25–34	192 (31.48)	23 (3.77)	1.23 (1.06, 2.07)	1.15 (0.06, 1.97)
>34	111 (18.20)	168 (27.53)	1	1
**Residence**				
Rural	367 (60.16)	145 (23.77)	3.11 (1.99, 4.83)	3.17 (0.11, 8.98)
Urban	44 (7.21)	54 (8.86)	1	1
**Ethnicity**				
Somali	392 (64.26)	170 (27.87)	1.46 (0.421, 5.053)	0.09 (0.003,3.090)
Amhara	10 (1.64)	17 (2.79)	3.00 (0.696,12.929)	0.05 (0.000,7.281)
Oromo	9 (1.47)	12 (1.97)	1	1
**Educational Status**				
No formal education	392 (64.26)	50 (8.20)	6.15 (3.506,10.773)	1.97 (1.583,4.496)∗
Formal education	19 (3.15)	149 (24.39)	1	1
**Marital status**				
Unmarried	21 (3.44)	70 (11.48)	0.10 (0.059, 0.168)	0.32 (0.002, 2.482)
Married	390 (63.93)	129 (21.15)	1	1
**Occupation**				
House wife	361 (59.18)	63 (10.33)	2.62 (0.602, 11.007)	4.37 (0.061, 17.432)
Merchant	16 (2.62)	45 (7.38)	1.33 (0.29, 6.073)	2.46 (0.096, 6.103)
Daily laborer	22 (3.61)	26 (4.26)	3.17 (0.977, 14.431)	1.06 (0.402, 2.196)
Gov't employer	12 (1.97)	70 (10.65)	1	1
**Wealth index**				
Low	138 (22.62)	61 (10.00)	1.57 (1.037,2.361)	2.26 (1.173,4.353)∗
Medium	153 (25.08)	55 (9.02)	1.92 (1.269,2.917)	2.54 (0.313,4.929)1
high	120 (19.67)	83 (13.61)	1	1
**ANC Visit**				
No	346 (56.72)	17 (2.79)	7.19 (1.481, 13.418)	6.16 (4.996,10.128)∗ 1
Yes	65 (10.66)	182 (29.83)	1	
**Gravidity**				
Primi para	51 (8.36)	16 (2.62)	12.05 (3.74, 21.86)	1.75 (0.091, 5.745)
Multi para	347 (56.89)	196 (32.13)	1	1
**Parity**				
Null parity	49 (2.95)	18 (11.80)	3.42 (2.976, 18.870)	2.70 (0.000, 4.153)
Low multi parity	409 (9.84)	61 (0.98)	1.66 (0.260, 4.552)	2.06 (0.038, 7.336)
Grand multi parity	46 (53.77)	27 (20.66)	1	1
**Knowledge about maternal nutrition**				
Poor	359 (58.85)	39 (6.39)	6.05 (1.721, 11.519)	4.94 (3.799, 8.748)∗
Good	52 (8.52)	160 (26.24)	1	1
**Attitude towards maternal nutrition**				
Negative	303 (49.67)	78 (12.79)	4.35 (3.038,6.235)	4.51 (1.588,12.806)∗
Positive	108 (17.71)	121 (19.83)	1	1

∗ = p < 0.05 CI = Confidence Interval, COR = Crude Odds Ratio, AOR = Adjusted Odds Ratio, ANC = Antenatal Care.

Pregnant women who had no formal education were 1.97 times more likely to have food taboos compared to their counterparts who had formal education [AOR = 1.97 (95%CI: 1.58, 4.49)]. Economically, pregnant women who had a low wealth index were 2.26 times more likely to have food taboos compared to pregnant women who had a high wealth index [AOR = 2.26 (95%CI: 1.17, 4.35)]. Pregnant women who had not antenatal care visits were 6.16 times more likely to have a food taboo compared to those who had antenatal care visits at least one time [AOR = 6.16 (95%CI: 4.99, 10.13)] (Table 4).

Pregnant women who had poor knowledge about maternal nutrition were 4.94 times more likely to have a food taboo compared to their counterparts who had higher nutrition knowledge levels [AOR = 4.94 (95%CI: 3.79, 8.75)]. Those who had a negative attitude towards maternal nutrition were 4.51 times more likely to have a food taboo compared to their counterparts who had positive attitude towards maternal nutrition [AOR = 4.51 (95%CI: 1.58, 12.81)] (Table 4).

## 4 Discussion

This study aimed to determine the prevalence of food taboos, the reasons, and factors that affect food taboos among pregnant women in Eastern Ethiopia. The study found that food taboos were significantly high among pregnant women.

The study findings showed that the prevalence of food taboos among pregnant women were 67.4%, which is higher compared to studies conducted in Hadya in 2015 [8], south Gondar in 2015 [4], and Addis Ababa city in 2019 [5] that showed the prevalence of food taboos were 27%, 45.6%, and 18.2%, respectively. This disparity might be due to the geographical differences in the study settings as our study was conducted in a pastoralist community of Eastern Ethiopia. The finding of this study was also greater when compared with studies conducted in other African countries such as Ghana in 2013 [14] and Nigeria in 2016 [15] that reported a prevalence of 37% and 48.6%**,** respectively. This might be due to differences in the sampling method in the case of Ghana and the difference in the level of knowledge of the study participants in the case of Nigeria.

On the other hand, the finding of this study is lower compared to studies reported in Bangalore [3], Malaysia [16], China [17], and Surendranagar [18] with a prevalence of food taboos 75%, 70.2%, 70.6%, and 77%, respectively. The probable reasons for this inconsistency may be the difference in the method of assessment in the case of Malaysia and China, the difference in age groups of participants in the case of Bangalore, and the difference in educational status in the case of Surendranagar. The difference in African and Asian countries may be another perceived difference in this finding.

In this study, pregnant women who had no formal education were nearly two-fold more likely to practice food taboos compared to their counterparts. This finding is consistent with study done in Hadya [8] and Nigeria [15]. This might be related to those non-educated mothers may not be clearly understood the nutrition information provided by health extension workers in the study area.

Pregnant women who had no antenatal care visits were more than six times more likely to practice food taboos compared to those having at least one antenatal care visits. This finding is supported by the study reported from Shashemene, Ethiopia [2]. This is because of that pregnant women having no antenatal care visits may not be getting nutrition education and counseling (NEC) from health care providers.

Pregnant women who had poor knowledge about maternal nutrition were nearly five times more likely to practice food taboos compared to their counterparts. This is consistent with findings in India, Iran, and China [13, 19, 20]. Women who had negative attitude towards maternal nutrition were 4.51 times more likely to have food taboos compared to their counterparts. This is also consistent with the study in Iran and China [19, 20]. This could be explained that women those who had poor knowledge and negative attitude towards maternal nutrition may not have formal education.

This study has important policy implications for our government, particularly the Minister of Health and local administers that food taboos compromising pregnant women’s nutritional status. There is an association between food taboos and absence of ANC visits. Hence, pregnant women must be encouraged to visit antenatal clinics during pregnancy. There is poor knowledge of pregnant women about maternal nutrition and the prevalence of food taboos were high. Thus, the information given by health extension workers about maternal nutrition must be revised to address the food taboos practice of pregnant women.

This finding indicated the need for implementation of food and nutrition policy which is drafted in December 2018, but not yet launched. This policy should include the promotion of varied diets and cultural change programs to address food taboos during pregnancy. This finding will also help the Federal Ministry of Health of Ethiopia to recognize food taboos as one of a challenge that should be addresses to improve the nutritional status of pregnant women in the country.

Regarding the limitations of the study**,** recall bias and **s**ocial desirability bias may be encountered. However, to minimize these expected biases, the researchers were used a private setting for interview, detail probing about the event and only female data collectors.

## 5 Conclusion

The finding showed that there is a high prevalence of food taboos. Low wealth index, not having antenatal care visits, having no formal education, poor knowledge of maternal nutrition, and negative attitudes towards maternal nutrition were the independent predictors of food taboos. To bring social and behavioral change powerful, community-based nutrition education, especially education about the risk of pregnancy-related food taboos, is recommended.

## Declarations

### Author contribution statement

Tesfa Mengie: Conceived and designed the experiments; Performed the experiments; Analyzed and interpreted the data; Contributed reagents, materials, analysis tools or data; Wrote the paper.

Yadeta Dessie, Gudina Egata: Conceived and designed the experiments; Analyzed and interpreted the data; Contributed reagents, materials, analysis tools or data.

Temesgen Muche, Samuel Derbie Habtegiorgis, Lemma Getacher: Performed the experiments; Analyzed and interpreted the data; Contributed reagents, materials, analysis tools or data; Wrote the paper.

### Funding statement

This research did not receive any specific grant from funding agencies in the public, commercial, or not-for-profit sectors.

### Data availability statement

Data will be made available on request.

### Declaration of interests statement

The authors declare no conflict of interest.

### Additional information

No additional information is available for this paper.
